# Left Atrial Appendage Closure for Atrial Fibrillation in the Elderly >75 Years Old: A Meta-Analysis of Observational Studies

**DOI:** 10.3390/diagnostics12123174

**Published:** 2022-12-15

**Authors:** Shaojie Han, Ruikun Jia, Shenyu Zhao, Juan Chan, Yixuan Bai, Kaijun Cui

**Affiliations:** Department of Cardiology, West China Hospital, Sichuan University, Chengdu 610041, China

**Keywords:** atrial fibrillation, left atrial appendage closure, meta-analysis, elderly, complication

## Abstract

Background: Left atrial appendage closure (LAAC) is an established therapy for patients with atrial fibrillation (AF); however, there is a limited understanding of LAAC in elderly patients (≥75 years old). We conducted a meta-analysis to investigate the procedural complications and long-term outcomes after LAAC in the elderly versus the non-elderly. Methods: We screened PubMed, EMBASE, Cochrane Library, and Web of Science. Procedural endpoints of interest included successful implantation LAAC rates, in-hospital mortality, major bleeding events, pericardial effusion/tamponade, stroke, and vascular access complications related to LAAC. Long-term outcomes included all-cause mortality, major bleeding events, and stroke/transient ischemic attack (TIA) during follow-up. Results: Finally, 12 studies were included in the analysis; these included a total of 25,094 people in the elderly group and 36,035 people in the non-elderly group. The successful implantation LAAC rates did not differ between the groups, while the elderly patients experienced more periprocedural mortality (OR 2.62; 95% CI 1.79–3.83, *p* < 0.01; I^2^ = 0%), pericardial effusion/tamponade (OR 1.39; 95% CI: 1.06–1.82, *p* < 0.01; I^2^ = 0%), major bleeding events (OR 1.32; 95% CI 1.17–1.48, *p* < 0.01; I^2^ = 0%), and vascular access complications (OR 1.34; 95% CI 1.16–1.55, *p* < 0.01; I^2^ = 0%) than the non-elderly patients. The long-term stroke/TIA rates did not differ between the elderly and the non-elderly at least one year after follow-up. Conclusions: Even though successful implantation LAAC rates are similar, elderly patients have a significantly higher incidence of periprocedural mortality, major bleeding events, vascular access complications, and pericardial effusion/tamponade after LAAC than non-elderly patients. The stroke/TIA rates did not differ between both groups after at least one-year follow-up.

## 1. Introduction

Atrial fibrillation (AF) has an increased incidence and prevalence with advancing age and is linked to a five-fold greater risk of ischemic stroke [1,2]. By 2050, there will be more than 5.6 million AF patients in the United States, with over half of them being 80 years of age or older [3]. In patients 80 years and older, the estimated annual risk of stroke from AF is 23.9% [4]. Although oral anticoagulation therapy is the mainstay for preventing thromboembolic cerebrovascular accidents, elderly people are more susceptible to bleeding incidents, and, as a result, these medications are frequently underutilized, mostly as a result of old age or the perceived high danger of bleeding problems, falls, or even polypharmacy [5,6,7]. Given its acceptable level of procedural safety and long-term effectiveness, percutaneous left atrial appendage closure (LAAC) is recognized as an alternative option for oral anticoagulants in patients with nonvalvular atrial fibrillation (NVAF) who want to prevent stroke, and current guidelines recommend LAAC for those whose long-term oral anticoagulation is deemed ineffective or contraindicated [6,8,9,10,11]. However, older patients tend to be more fragile and have a co-morbidity burden, both of which frequently coexist and affect clinical outcomes [12,13].

Due to the relatively small sample sizes of the existing studies, the results of LAAC for older individuals with AF remain inconsistent [14,15,16,17]. To address these problems, we carried out a meta-analysis of research examining the procedural safety and long-term effectiveness of LAAC for elderly patients with AF.

## 2. Methods

The Preferred Reporting Items for Systematic Reviews and Meta-Analyses criteria were followed when conducting the study [18].

### 2.1. Search Strategies

Up to October 2021, a thorough search of the literature was conducted in PubMed, Web of Science, Cochrane Library, and Embase databases. Search terms included (“left atrial appendage” OR “auricula sinistra” OR “left atrium appendage” OR “atrial appendage”) AND (elderly OR “older patients” OR octogenarian OR nonagenarian) AND (“atrial fibrillation”). Through a manual search of secondary materials, the references of initially found articles, reviews, and comments were found. All references were downloaded to consolidate, eliminate duplicates, and conduct further research. Detailed search strategies are provided in Supplemental File S1.

### 2.2. Selection and Data Abstraction

The search results were assessed by two blinded, independent writers (S.H. and R.J.), who included papers if they matched the following requirements: (1) the inclusion criteria were human studies with a parallel design, (2) compared elderly patients (over 75 years old) versus non-elderly patients undergoing LAAC for AF, and (3) at least one procedure-related complication was reported. With regards to sample size, follow-up time, and language, we had no limitations. Reviews, case studies, conference papers, and animal trials were all omitted from the study. Two reviewers separately evaluated article titles and abstracts to exclude papers that were not relevant. Disagreements were addressed by consensus and, if needed, the consultation of a third reviewer. The following information was gathered from eligible studies: (1) design of the research; (2) primary/secondary outcome; and (3) mean follow-up time and baseline characteristics. The Risk of Bias Assessment Tool for Nonrandomized Studies (RoBANS) was used to assess the risk of bias [19].

### 2.3. Outcome Measures

Successful implantation LAAC rates and procedural-related complications were assessed, including periprocedural mortality, major bleeding events, pericardial effusion/tamponade, stroke, and vascular access complications. We also assessed long-term outcomes, including all-cause mortality, stroke/ transient ischemic attack (TIA), and major bleeding events. Device success was defined as correct deployment and implantation of the respective LAA occlude types in the included literature.

### 2.4. Statistical Analysis

Given the expected between-study heterogeneity, a meta-analysis was conducted using a random effects model for all outcomes. Dichotomous variables were investigated using the Mantel-Haenszel method. The odds ratio (OR) and 95% confidence interval (CI) were determined. To examine heterogeneity, the Cochran Q and I^2^ statistics were utilized. We defined moderate or high heterogeneity as an I^2^ of more than 50%. If more than 10 studies were included, a funnel plot was used to measure publication bias. We carried out a series of sensitivity analyses to determine the contribution of each study to the pooled estimate by deleting one study at a time. A *p*-value of <0.05 was considered statistically significant. The Nordic Cochrane Centre’s Review Manager Version 5.3 software was utilized to carry out the overall effect analysis.

## 3. Results

**Figure 1:**
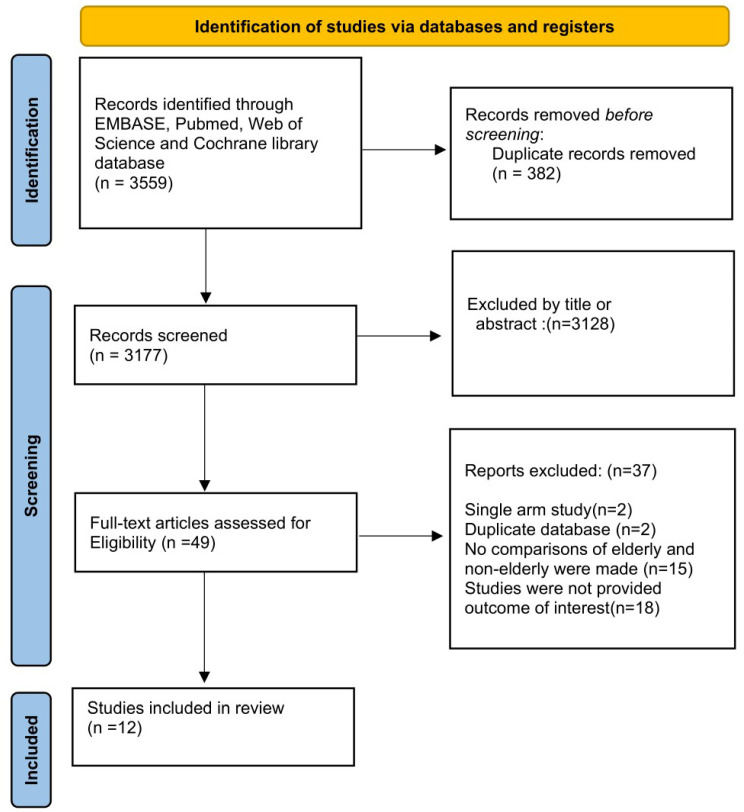
Summary of electronic search and included/excluded studies.

Initial results from our search method returned 3559 possibly related articles (Figure 1). After the exclusion of 382 duplicate articles, 3177 publications were screened by titles or abstracts. According to article categories, methodology, and outcomes of interest based on reading the title or abstract, a total of 3128 publications were eliminated because they did not match the inclusion criteria. The whole read consisted of 49 articles. Due to the fact that there was only a single-arm study, two studies were removed. Two studies were excluded because they were from the same database. Fifteen studies were excluded because they were not grouped based on age. Eighteen studies were excluded because no outcomes of interest were provided. Finally, 12 articles—11 observational studies and 1 analysis matched on propensity scores—were used in our study [14,15,16,17,20,21,22,23,24,25,26,27]. The characteristics of the study are shown in Table 1. Supplementary Table S1 provides an overview of the bias risk associated with RoBANS in each study. The total population in our study was 61,129 patients (25,094 elderly patients, 41.1%).

### 3.1. Successful Implantation LAAC Rates and Peri-Procedural Complications

**Figure 2:**
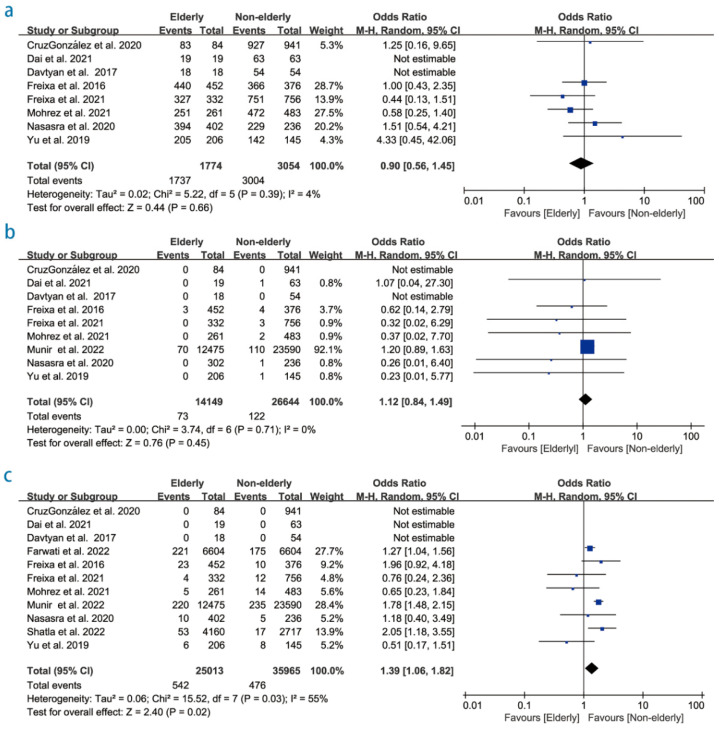
Forest plot showing the effect of left atrial appendage closure on (**a**) successful implantation LAAC rates, (**b**) stroke, and (**c**) pericardial effusion/tamponade [14,15,16,17,20,21,22,23,24,25,26,27].

Eight studies (4828 patients) reported successful implantation LAAC rates. A total of 1737 patients had successful implantation LAAC among 1774 patients (97.9%) in the elderly group, compared with 3004 LAAC among 3054 patients (98.3%) in the non-elderly arm. The pooled estimate showed no statistically significant difference between the two groups (OR:0.90; 95% CI:0.56–1.45; *p* = 0.66; I^2^ = 4%; Figure 2a). All procedure-related complications are listed in Supplementary Table S2. Total periprocedural mortality, major bleeding events, pericardial effusion/tamponade, stroke, and vascular access complications associated with the procedure were individually sought. There were no differences in stroke between the elderly and the non-elderly (OR 1.12; 95% CI 0.84–1.49, *p* = 0.45; I^2^ = 0%; Figure 2b). The overall meta-analysis indicated that the elderly patients were at 39% higher risk for pericardial effusion/tamponade (OR: 1.39; 95% CI: 1.06–1.82, *p* = 0.02; I^2^ = 0%; Figure 2c).

**Figure 3:**
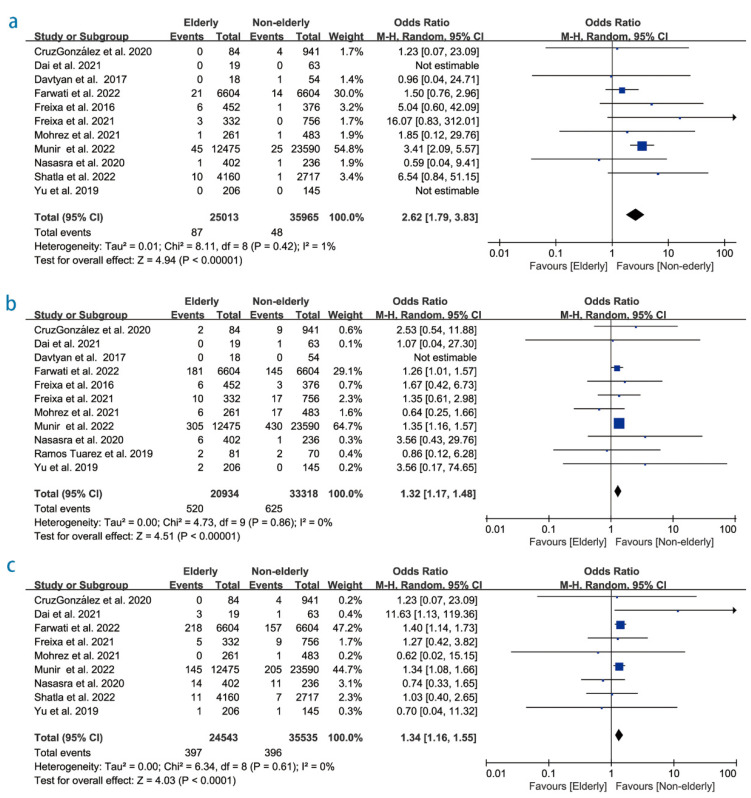
Forest plot showing the effect of left atrial appendage closure on (**a**) periprocedural mortality, (**b**) major bleeding events, and (**c**) vascular access complication [14,15,16,17,20,21,22,23,24,25,26,27].

Additionally, our study showed that the elderly group’s periprocedural mortality was statistically significantly higher than that of the non-elderly group (OR 2.62; 95% CI 1.79–3.83, *p* < 0.01; I^2^ = 0% Figure 3a) with regards to major bleeding events (OR 1.32; 95% CI 1.17–1.48, *p* < 0.01; I^2^ = 0%; Figure 3b) and vascular access complications (OR 1.34; 95% CI 1.16–1.55, *p* < 0.01; I^2^ = 0%; Figure 3c).

### 3.2. Outcomes at Follow-Up

**Figure 4:**
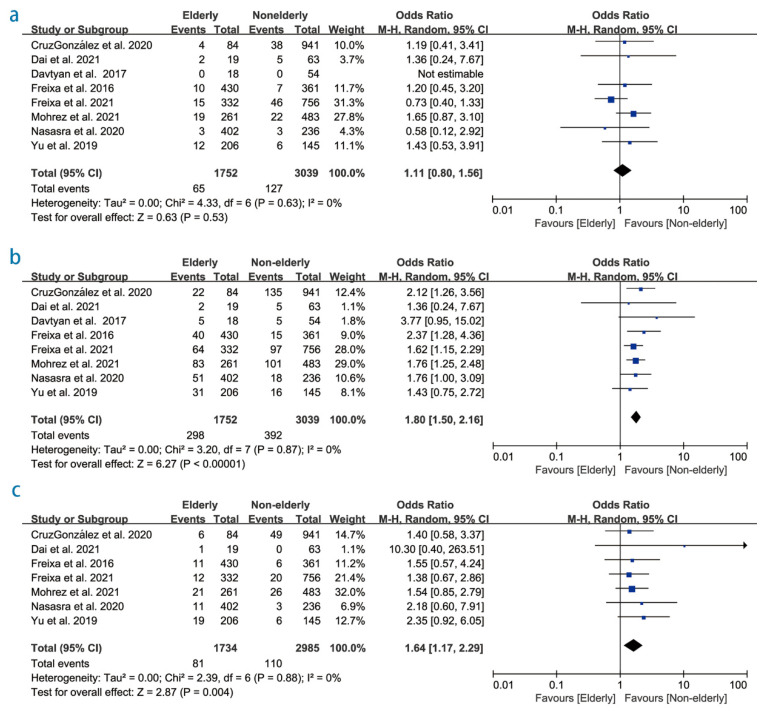
Forest plot showing the effect of left atrial appendage closure on (**a**) the risk of stroke/transient ischemic attack, (**b**) all-cause death, and (**c**) major bleeding events in the follow up time [14,17,20,22,23,25,26].

A total of eight studies reported follow-up outcomes ranging from 12 to 24 months. An amount of 4791 patients assessed ischemic stroke/TIA. The risk of stroke/TIA was not significantly increased in the older group (OR 1.11; 95% CI 0.80–1.56; *p* = 0.53; I^2^ = 0%; Figure 4a). Sensitivity analysis revealed that no single study had a significant effect on the combined OR. Eight studies reported all-cause mortality during follow-up, and the older group had higher all-cause mortality during follow-up than the non-older age group (OR 1.80; 95% CI 1.50–2.16; *p* < 0.01; I^2^ = 0%; Figure 4b). Only four studies reported the risk of cardiovascular death, and there was no statistically significant difference between the two groups. Seven studies (4719 patients) reported major bleeding events during the follow-up. There were 81 events among 1734 patients (4.7%) in the elderly group and 110 events among 2985 patients (3.7%) in the non-elderly group. The pooled estimate showed a statistically significant difference between the two groups (OR: 1.64; 95% CI: 1.17–2.29; *p* <0.01; I^2^ = 0%; Figure 4c). Sensitivity analysis revealed that no single study had a significant effect on the combined OR (Supplementary Table S3). The incidence of stroke/TIA, bleeding, and all-cause mortality during the follow up time in the elderly group were 2.1% per year, 2.9% per year, and 11.8% per year, while in the non-elderly were 2.2% per year, 1.9% per year, and 7.5% per year, respectively.

### 3.3. Publication Bias and Sensitivity Analysis

Considering the number of studies, only periprocedural mortality, major bleeding events, and pericardial effusion/tamponade were evaluated for publication bias (Supplementary Figures S1–S3). We conducted a sensitivity analysis by eliminating one study at a time to determine how each one affected the outcomes. Supplementary Table S3 in the Supplementary Material provides an overview of the results of the sensitivity analysis.

## 4. Discussion

The following are the key conclusions of this meta-analysis: (1) the successful implantation LAAC rates of LAAC were comparable for older and non-elder patients. (2) We found that elderly patients with LAAC had a significantly higher incidence of periprocedural mortality, major bleeding events, pericardial effusion/tamponade, and vascular access complications during the perioperative period compared to non-elderly patients. There was no difference in stroke between the elderly and non-elderly patients during the perioperative period. (3) At the follow-up of at least one year after LAAC, the elderly group had a higher risk of all-cause mortality and major bleeding events. However, the risk of stroke/TIA was comparable in both groups.

### 4.1. Perioperative Safety

Given that elderly patients with AF are more likely to experience major bleeding, as well as cardioembolic events, LAAC is a desirable alternative. It was necessary to investigate the effectiveness and safety of this procedure in this particular group because elderly patients are more brittle and prone to complications. There have been numerous randomized trials comparing LAAC with medical therapy for patients with AF, but in the PROTECT AF and PREVAIL trials, no specific analysis was performed for elderly patients, and 41% and 52% of patients were over 75 years old in these trials, respectively [8,28].

Our meta-analysis showed that elderly patients had a higher risk of periprocedural mortality. Although only one study in our included study found a greater risk of death in the elderly group [14], this study was excluded by sensitive analysis, and the statistical results remained statistically significant. Only one study clearly gave the cause of death: one procedure-related death occurred the next day [17]. Other studies did not explain the specific cause of death. However, LAAC is usually an elective surgery, and patients generally do not have severe acute illnesses when they are readmitted to the hospital. We speculate that periprocedural mortality may be related to complications caused by surgery.

In addition, our study found more vascular access complications and major bleeding events in elderly patients than non-elderly patients. Hirata et al. found that the age of patients with AF affected the LAA structure [29]. Therefore, we thought that some characteristics, such as left atrial size and LAA structure, may also have important implications for complications. Gastrointestinal bleeding dominates in our included studies. In the study of Ramos Tuarez et al., major bleeding patients did not rebleed after changing the anticoagulation regimen [21]. In the study by Dai et al., there was one case of gastrointestinal bleeding in the elderly group, which improved after treatment [24].

The successful implantation LAAC rates were comparable for the elderly and non-elder, but device success was defined as correct deployment and implantation of the respective LAA occlude in the included literature. A patient may not benefit from “successful implantation”. An amount of 125 patients (15.5%) died within the first year of LAAC in the study of Mesnier et al. In the multivariable analysis, a factor associated with early death after LAAC was older age (HR: 1.03; 95% CI: 1.01–1.06 per year; *p* = 0.01) [30]. In addition, one study concluded that advanced age is associated with readmission after LAAC [31]. We also calculated the prevalence of comorbid conditions, such as coronary artery disease, congestive cardiac failure, and diabetes mellitus, between the two groups. Compared with the non-elderly group, we found that the elderly group had higher CHA2DS2-VASc scores and prevalence of coronary heart disease. We speculate that a polymorbid patient population is the cause of the relatively high periprocedural mortality rates. In our study, the elderly group had more females (43.6% vs. 40.4%, *p* < 0.01). In the study of Darden et al., women have a significantly higher risk of in-hospital adverse events after LAAC, including pericardial effusion requiring drainage, major bleeding, and or death compared with men [32]. A meta-analysis also concluded that women have a significantly higher incidence of pericardial complications, major bleeding, and vascular complications following LAAC [33].

Early LAAC was associated with poor success rates (approximately 90%) and higher perioperative complication rates (approximately 8.4%) [28]. However, due to improvements in surgical techniques and the adoption of standardized procedures, the success rate of surgeries has progressively grown to 98.5%, while major perioperative adverse events have fallen to 2.7% [9]. Our study consisted mainly of patients with LAAC before 2018. With the increase in the number of LAAC, the likelihood of adverse events occurring during the perioperative period normally decreases, even in elderly patients [34].

### 4.2. Efficacy during Follow-Up

After examining the safety of LAAC in older patients, the next step was to evaluate its long-term effectiveness. Patients with AF have a significantly higher risk of ischemic stroke, and there is evidence that this risk increases with advancing age [4]. A total of eight studies reported follow-up outcomes ranging from 12 to 24 months. Compared with the non-elderly group, the elderly group did not have a significantly higher rate of stroke/TIA, which highlighted that LAAC seems to be effective in the long term. According to Mohrez et al., the observed stroke rate among octogenarians was 41% lower than what was expected [25]. 

In contrast with the incidence of ischemic strokes, the incidence of major bleeding events was significantly higher in older patients. In the elderly group, the annual major bleeding risk was also higher than that in the non-elderly group (2.9% vs. 19% *p* = 0.05). However, we think it has little to do with the operation of the LAAC. During the follow-up, seven articles reported major bleeding events, and one of them detailed the treatment of bleeding events. Gastrointestinal bleeding still accounted for more than 50% [16,25]. In the study by Dai et al., one patient in the elderly group had gastrointestinal bleeding during the perioperative period and was given rivaroxaban 10 mg/d. One patient died of cerebral hemorrhage four months after surgery [24]. LAAC has been linked to significantly fewer bleeding events than oral anticoagulation, according to randomized trials and propensity score matched studies [35,36]. Elderly patients with AF usually have higher HAS-BLED scores. The observed bleeding rate in octogenarians was 10% lower than the HAS-BLED score’s predicted rate. The majority of bleeding events involved the gastrointestinal system, and more than one-third of major bleeding events occurred within the first 30 days [16]. Some research analyses show that gastrointestinal bleeding was the most typical readmission reasons after LAAC in the first 30 days [37,38]. We believe that the rate of major bleeding observed may point to the need for elderly patients to receive customized antithrombotic treatment based on their age and the type of bleeding they experience following LAAC.

Mortality increased gradually as patients aged, as expected. An amount of 17.0% of patients in the elderly group passed away during the two-year follow up. This outcome makes sense, given that elderly patients have a higher prevalence of comorbid conditions, such as coronary artery disease and renal impairment. In this regard, the key to delivering a higher clinical benefit and avoiding pointless procedures in very ill patients may lie in superior patient selection. Some meta-analyses concluded that elderly patients’ long-term catheter ablation for AF effectiveness was equivalent to that of non-elderly patients’, but the incidence of operational complications was higher in elderly patients [39,40]. These findings imply that increased caution and concern for complications should be used when performing invasive cardiac procedures on elderly patients.

## 5. Limitations

This study has a number of drawbacks. First, we lacked access to individual patient data and could only utilize accessible summary data from published research. Second, all studies were observational cohort studies, but we may set this aside, as thy are the only available data. Each study had a different age distribution. However, according to our thorough search, there are no randomized controlled studies on this topic. Therefore, this analysis included the “best-available” data. Third, patients from the United States who were undergoing LAAC were included in three studies. Two studies recruited patients from the National Inpatient Sample (NIS), but their age groupings were different, 75 and 80 years old [14,27]. One study included patients from the National Readmission Database (NRD) [26]. Although the objectives and eligibility requirements for these databases differ, it still raises questions about the possibility that some patients may have duplicated in our study. It is necessary to conduct large and randomized studies to confirm these results.

## 6. Conclusions

Even though procedural success rates are similar, elderly patients have a significantly higher incidence of periprocedural mortality, major bleeding events, vascular access complications, and pericardial effusion/tamponade after LAAC compared to the non-elderly patients. There is no difference in the long-term stroke/TIA rates between elderly and non-elderly patients.

## Figures and Tables

**Table 1:** Characteristics of the 12 included studies.

Study	Country	Study Design	Type of Occluder	Groups	Follow-Up	Sample, N	Male, N (%)	Mean Age, Years	CHA2DS-2VASc Score,	HTN, N (%)	DM, N (%)	Stroke/TIA, N (%)	CAD, N (%)
Freixa et al. (2016).	European	Retrospective multicenter study	ACP	a	16.8 m	452	248 (54.9)	81.2 ± 4.1	5.1 ± 1.4	395 (87.4)	134 (29.8)	172 (38.1)	NR
				b	16.8 m	376	255 (67.8)	68.4 ± 5.6	3.9 ± 1.5	327 (87.0)	123 (32.7)	150 (39.9)	NR
Davtyan et al. (2017).	Russia	single-centre, retrospective study	ACP and Watchman	a	12 m	18	4 (22.2)	77.8 ± 3.1	5.3 ± 1.6	NR	NR	12 (66.7)	6 (33.3)
				b	12 m	54	20 (37.1)	65.7 ± 5.7	4.8 ± 1.5	NR	NR	32 (58.6)	8 (15.1)
Ramos Tuarez, et al. (2019).	USA	single- centre, retrospective study	Watchman	a	24 m	81	56 (69.1)	NR	NR	73 (90.1)	32 (39.5)	20 (24.7)	NR
				b	24 m	70	47 (68.1)	73.2 ± 4.3	4.2 ± 1.4	61 (87.1)	30 (42.9)	25 (35.7)	NR
Yu et al. (2019)	Germany	single- centre, retrospective study	Watchman	a	21.6 m	206	126 (61.2)	80.1 ± 3.9	4.5 ± 1.4	166 (80.6)	59 (28.6)	39 (18.9)	116 (56.3)
				b	23.8 m	145	108 (74.5)	67.7 ± 6.5	3.0 ± 1.3	115 (79.3)	40 (27.6)	22 (15.2)	68 (46.9)
CruzGonzález et al. (2020).	Spain.	Retrospective multicenter study	Watchman	a	732 d	84	38 (45.2)	87.4 ± 2.3	5.2 ±1.1	71 (84.5)	17 (20.2)	19 (22.6)	NR
				b	732 d	941	576 (61.2)	72.2 ± 8.1	4.4 ±1.6	817 (86.8)	287 (30.5)	294 (31.2)	NR
Nasasra et al. (2020).	Germany	Retrospective multicenter study	Watchman, ACP and Amulet	a	12 m	402	238 (59.2)	80.6 ± 3.8	5.1 ± 1.3	NR	138 (34.3)	103 (25.7)	215 (53.5)
				b	12 m	236	152 (64.4)	68.0 ± 6.6	3.6 ± 1.5	NR	79 (33.5)	86 (36.4)	77 (32.6)
Dai et al. (2021).	China	single- centre, retrospective study	Amulet	a	24 m	19	12 (63.3)	81.2 ± 3.6	4.7 ± 1.3	7 (36.8)	2 (10.5)	11 (57.9)	7 (36.8)
				b	24 m	63	51 (81.0)	64.8 ± 8.0	3.6 ± 1.2	26 (41.3)	17 (27.0)	47 (74.6)	14 (22.2)
Mohrez et al. (2021)	Germany	Retrospective multicenter study	ACP, Amulet and Watchman	a	1.7 y	261	138 (52.9)	84.0 ± 3.0	5.2 ± 1.2	236 (90.4)	80 (30.7)	NR	158 (60.5)
				b	2.3 y	483	330 (68.3)	70.4 ± 7.8	4.3 ± 1.7	434 (89.9)	159 (32.9)	NR	236 (48.9)
Freixa et al. (2021).	Germany	Retrospective multicenter study	Amulet	a	2 y	491	309 (63)	76.0 ± 3.0	4.3 ± 1.5	412 (84)	NR	191 (39)	128 (26)
				b	2 y	332	206 (62)	84.0 ± 3.0	4.8 ± 1.3	279 (84)	NR	133 (40)	93 (28)
Farwati et al. (2022).	USA	Retrospective multicenter study	Watchman	a	NR	6604	3753 (56.8)	84 (81-86)	4 (4–6)	5694 (86.2)	1841 (27.9)	1640 (24.8)	NR
				b	NR	6604	3775 (57.2)	73 (69–77)	4 (3–5)	5757 (87.2)	1871 (28.3)	11,554 (23.5)	NR
Shatla et al. (2022).	USA	Retrospective multicenter study	Watchman	a	NR	4160	2356 (56.7)	81.1 ± 4.3	NR	2356 (56.6)	1284 (30.9)	1073 (25.8)	707 (17.0)
				b	NR	2717	1635 (60.2)	68.2 ± 5.6	NR	1625 (59.8)	1140 (42.0)	755 (27.8)	362 (13.3)
Munir et al. (2022).	USA	Retrospective multicenter study	Watchman	a	NR	12,475	6980 (56.0)	NR	4 (3–5)	10,635 (85.3)	1910 (15.3)	NR	965 (7.7)
				b	NR	23,590	14,065 (59.6)	NR	3 (3–4)	20,280 (86.0)	5050 (21.4)	NR	1825 (7.7)

HTN: hypertension; DM: diabetes mellitus; TIA, transient ischemic attack; CAD: coronary artery disease; ACP: AMPLATZER cardiac plug; NR: not reported; a: elderly group; b: non-elderly group.

## Data Availability

This research is a meta-analysis, and all data have been uploaded along with the Supplementary Materials.

## Supplementary Materials

The following supporting information can be downloaded at: https://www.mdpi.com/article/10.3390/diagnostics12123174/s1, Supplemental File S1: Search Strategies; Figure S1: Funnel Plot of Studies Pooled for In-hospital Mortality; Figure S2: Funnel Plot of Studies Pooled for Pericardial Effusion/Tamponade; Figure S3: Funnel Plot of Studies Pooled for Major Bleeding Events; Table S1: Clinical Outcomes; Table S2: The Risk of Bias Assessment tool for Non-randomized Studies (RoBANS); Table S3: Sensitivity Analysis.
