# Supplementary material for: Left Atrial Appendage Closure for Atrial Fibrillation in the Elderly >75 Years Old: A Meta-Analysis of Observational Studies
Source: Diagnostics (Basel). 2022 Dec 15;12(12):3174. doi: 10.3390/diagnostics12123174 (PMC9777302; doi:10.3390/diagnostics12123174)
Supplement: Supplementary file 1 [file diagnostics-12-03174-s001.zip › diagnostics-2070316-supplementary.pdf]

Supplemental Material

1. Supplemental file S1

Search Strategies

#1 "Atrial Fibrillation"[Mesh] OR "atrial fibrillation"[Title/Abstract]

#2 "Atrial Appendage"[Mesh] OR "Left Atrial Appendage"[Title/Abstract] OR "auricula sinistra"[Title/Abstract] OR "left atrium appendage"[Title/Abstract]

#3 elderly [All Fields] OR "older patients"[All Fields] OR octogenarian [All Fields] OR nonagenarian [All Fields]

#4 #1 AND #2 AND #3

2. Supplemental figures

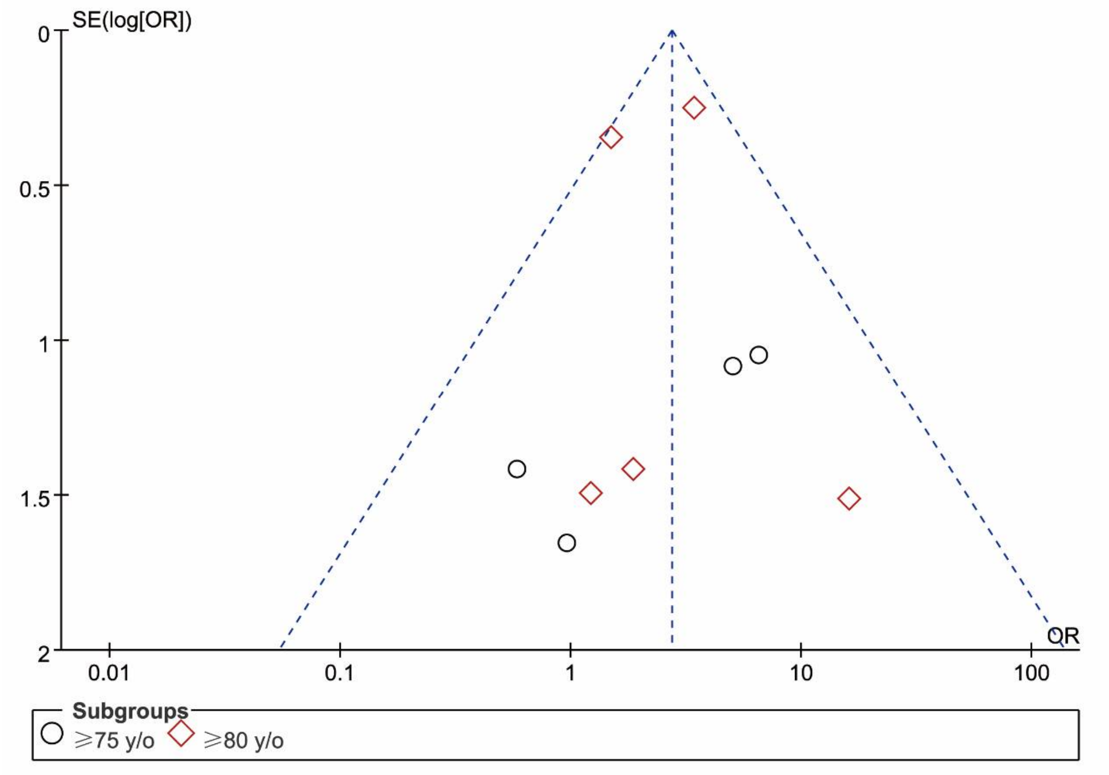

Figure S1. Funnel plot of studies pooled for in-hospital mortality.

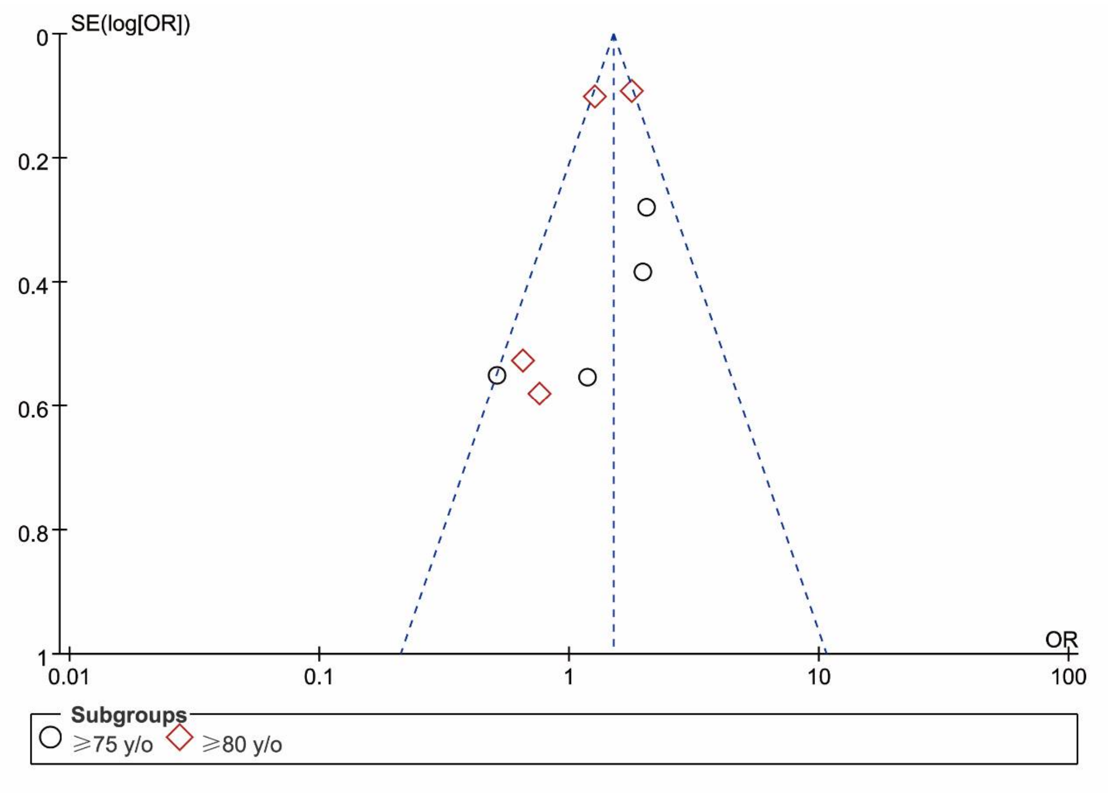

Figure S2. Funnel plot of studies pooled for pericardial effusion/tamponade.

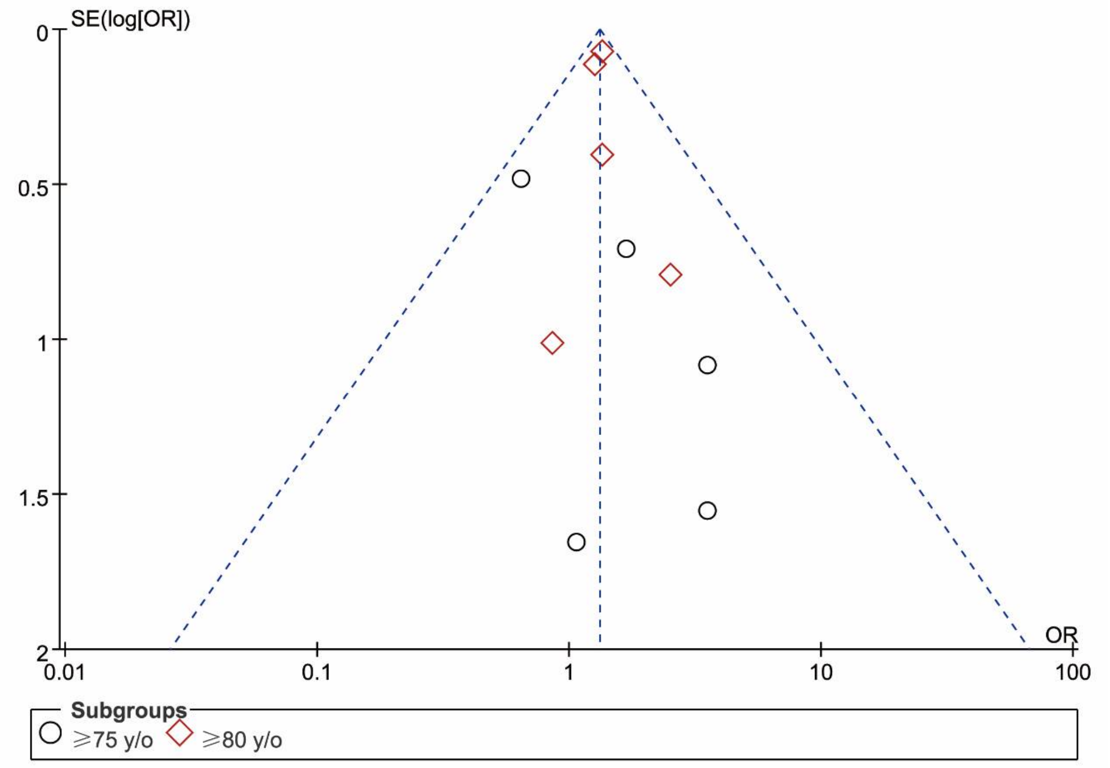

Figure S3. Funnel plot of studies pooled for major bleeding events.

3. Supplemental tables

Table S1. Clinical Outcomes of Included Studies.

									Outcomes of follow-up		
Study	groups	Success, Total(n)	In-hospital mortality, Total (n)	Major bleeding events, Total (n)	Pericardial Effusion/ Tamponade, Total (n)	Stroke, Total, (n)	Vascular complication, Total(n)	Acute kidney injury, Total (n)	All-cause mortality, Total (n)	Stroke/ TIA, Total (n)	Major bleeding events, Total (n)
Freixa et al. (2016).	A	440/452	6/452	6/452	23/452	3/452	NR	NR	40/430	10/430	11/430
	B	366/376	1/376	3/376	10/376	4/376	NR	NR	15/361	7/361	6/361
Davtyan et al. (2017)	A	18/18	0/18	0/18	0/18	0/18	NR	NR	5/18	0/18	NR
	B	54/54	1/54	0/54	0/54	0/54	NR	NR	5/54	0/54	NR
RamosTuarez, et al. (2019).	A	NR	NR	2/81	NR	NR	NR	3/70	NR	NR	NR
	B	NR	NR	2/70	NR	NR	NR	3/71	NR	NR	NR
Yu et al. (2019)	A	205/206	0/206	2/206	6/206	0/206	1/206	NR	31/206	12/206	19/206
	B	142/145	0/145	0/145	8/145	1/145	1/205	NR	16/145	6/145	6/145
CruzGonzález et al. (2020).	A	83/84	0/84	2/84	0/84	0/84	0/84	NR	22/84	4/84	6/84
	B	927/941	4/941	7/941	0/941	0/941	4/941	NR	135/941	38/941	49/941
Nasasra et al. (2020).	A	392/402	1/402	6/402	10/402	0/402	14/402	NR	51/402	3/402	11/402
	B	229/236	1/236	1/236	5/236	1/236	11/236	NR	18/236	3/236	3/236
Dai et al. (2021).	A	19/19	0/19	0/19	0/19	0/19	3/19	NR	2/19	2/19	1/19
	B	63/63	0/63	1/63	0/63	1/63	1/63	NR	5/63	5/63	0/63
	A	251/261	1/261	6/261	5/261	0/261	0/261	2/261	83/261	19/261	21/261

Mohrez et al. (2021)	B	472/483	1/483	17/483	14/483	2/483	1/483	9/483	101/483	22/483	26/483
Freixa et al. (2021).	A	327/332	3/332	10/332	4/332	0/332	5/332	NR	64/332	15/332	12/332
	B	751/756	0/756	17/756	12/756	3/756	9/756	NR	97/756	46/756	20/756
Farwati et al. (2022).	A	NR	21/6604	181/6604	221/6604	21/6604[a]	218/6604	180/6604	NR	NR	NR
	B	NR	14/6604	145/6604	175/6604	14/6604[a]	157/6604	177/6604	NR	NR	NR
Shatla et al. (2022).	A	NR	10/4160	NR	53/4160	10/4160[a]	11/4160	NR	NR	NR	NR
	B	NR	1/2717	NR	17/2717	1/2717[a]	7/2717	NR	NR	NR	NR
Munir et al. (2022).	A	NR	45/12475	340/12475	220/12475	70/12475	145/1247	NR	NR	NR	NR
	B	NR	25/23590	615/23590	235/23590	110/23590	205/23590	NR	NR	NR	NR

TIA: Transient ischemic attack; NR: Not reported; a: included not only stroke but also TIA. A: elderly group; B:Non-elderly group.

Table S2. The Risk of Bias Assessment tool for Non-randomized Studies (RoBANS).

Study	The selection of participants	Confounding variables	Measurement of exposure	Blinding of outcome assessments	Incomplete outcome data	Selective outcome reporting
Freixa et al. (2016).	Low risk	High risk	Low risk	High risk	Low risk	Low risk
Davtyan et al. (2017).	Low risk	High risk	Low risk	High risk	Low risk	High risk
Ramos Tuarez, et al. (2019).	Low risk	High risk	Low risk	High risk	Low risk	Low risk
Yu et al. (2019)	Low risk	Unclear risk	Low risk	High risk	Low risk	Low risk
CruzGonzález et al. (2020).	Low risk	High risk	Low risk	High risk	Low risk	Low risk
Nasasra et al. (2020).	Low risk	High risk	Low risk	High risk	Low risk	Low risk
Dai et al. (2021).	Low risk	High risk	Low risk	High risk	Low risk	Low risk
Mohrez et al. (2021)	Low risk	Low risk	Low risk	High risk	Low risk	Low risk
Freixa et al. (2021).	Low risk	Low risk	Low risk	High risk	Low risk	Low risk
Farwati et al. (2022).	Low risk	Low risk	Low risk	High risk	Unclear risk	Low risk
Shatla et al. (2022).	Low risk	Low risk	Low risk	High risk	Unclear risk	Low risk
Munir et al. (2022).	Low risk	High risk	Low risk	High risk	Unclear risk	Low risk

Table S3: Sensitivity Analysis

Study Excluded	Odds Ratio (95% Confidence Interval)	*p*-value	I^2(%)
**Success rate**			
Freixa et al. 2016	0.89 (0.46 – 1.71)	0.73	22

Yu et al. 2019	0.84 (0.52 – 1.34)	0.46	0
Nasasra et al. 2020.	0.79 (0.47 – 1.32)	0.36	0
CruzGonzález et al. (2020).	0.89 (0.52 – 1.55)	0.69	22
Freixa et al. (2021).	1.00(0.61 – 1.65)	0.98	0
Mohrez et al. 2021	1.06(0.62 – 1.82)	0.84	0
**In-hospital mortality**			
Freixa et al. 2016	2.49 (1.57 – 3.94)	<0.001	9
Davtyan et al. 2017	2.61 (1.67 – 4.10)	<0.001	10
CruzGonzález et al. 2020	2.61 (1.64 – 4.14)	<0.001	11
Nasasra et al. 2020	2.70 (1.87 – 3.92)	<0.001	0
Freixa et al. 2021	2.56 (1.77 – 3.71)	<0.001	0
Mohrez et al. 2021	2.58 (1.59 – 3.71)	<0.001	0
Shatla et al. 2022	2.50 (1.65 – 3.78)	<0.001	4
Farwati et al. 2022	3.33 (2.15 – 5.16)	<0.001	0
Munir et al. 2022	1.88 (1.08 – 3.28)	<0.001	0
**Stroke**			
Freixa et al. 2016	1.14 (0.85 – 1.53)	0.47	0
Yu et al. 2019	1.13 (0.85 – 1.51)	0.40	0
Nasasra et al. 2020	1.13 (0.85 – 2.52)	0.40	0
Dai et al. 2021	1.12 (0.84 – 1.49)	0.45	0
Freixa et al. 2021	1.13 (0.85 – 1.51)	0.40	0
Munir et al. 2022	0.47 (0.17 – 1.32)	0.15	0
**Pericardial effusion/tamponade**			
Freixa et al. 2016	1.33 (0.99 – 1.78)	0.06	60
Yu et al. 2019	1.48 (1.16 – 1.90)	0.002	49
Nasasra et al. 2020	1.39 (1.05 – 1.86)	0.02	61
Mohrez et al. 2021	1.46 (1.12 – 1.90)	0.005	54
Freixa et al. 2021	1.43 (1.09 – 1.89)	0.010	57
Shatla et al. 2022	1.30 (0.96 – 1.75)	0.09	58
Farwati et al. 2022	1.19 (0.75 – 1.88)	0.47	53
Munir et al. 2022	1.15 (0.84 – 1.57)	0.39	20
**Major bleeding events**			
Freixa et al. 2016	1.31 (1.17 – 1.48)	<0.001	0
Yu et al. 2019	1.31 (1.17 – 1.48)	<0.001	0
Ramos Tuarez et al. 2019	1.32 (1.17 – 1.49)	<0.001	0
Nasasra et al. 2020	1.31(1.16 – 1.48)	<0.001	0
CruzGonzález et al. 2020	1.31 (1.16 – 1.48)	<0.001	0
Dai et al. 2021	1.31 (1.17 – 1.48)	<0.001	0

Mohrez et al. 2021	1.33 (1.18 – 1.50)	<0.001	0
Freixa et al. 2021	1.32 (1.17 – 1.50)	<0.001	0
Munir et al. 2022	1.26 (1.03 – 1.54)	0.03	0
Farwati et al. 2022	1.34 (1.16 – 1.55)	0.97	0
**Vascular access complication**			
Dai et al. 2021	1.33 (1.15 – 1.53)	<0.001	0
Mohrez et al. 2021	1.34 (1.16 – 1.55)	<0.001	0
Nasasra et al. 2020	1.37 (1.18 – 1.58)	<0.001	0
Shatla et al. 2022	1.35 (1.17 – 1.56)	<0.001	0
Yu et al. 2019	1.34 (1.16 – 1.55)	<0.001	0
CruzGonzález et al. 2020	1.34 (1.16 – 1.55)	<0.001	0
Farwati et al. 2022	1.29 (1.06 – 1.57)	0.01	0
Freixa et al. 2021	1.34 (1.16 – 1.55)	<0.001	0
Munir et al. 2022	1.34 (1.11 – 1.62)	0.001	0
**All-cause mortality in the follow up time**			
CruzGonzález et al. 2020	1.76 (1.44 – 2.14)	<0.001	0
Dai et al. 2021	1.80 (1.50 – 2.17)	<0.001	0
Davtyan et al. 2017	1.77 (1.47 – 2.13)	<0.001	0
Freixa et al. 2016	1.75 (1.44 – 2.12)	<0.001	0
Freixa et al. 2021	1.87 (1.51 – 2.32)	<0.001	0
Mohrez et al. 2021	1.81 (1.46 – 2.25)	<0.001	0
Nasasra et al. 2020	1.80 (1.48 – 2.19)	<0.001	0
Yu et al. 2019	1.83 (1.52 – 2.22)	<0.001	0
**Stroke/TIA during in the follow up time**			
CruzGonzález et al. 2020	1.11 (0.78 – 1.57)	0.58	0
Dai et al. 2021	1.10 (0.79 – 1.55)	0.57	0
Davtyan et al. 2017	1.11 (0.80 – 1.56)	0.53	0
Freixa et al. 2016	1.11 (0.77 – 1.57)	0.59	0
Freixa et al. 2021	1.35 (0.90 – 2.02)	0.15	0
Mohrez et al. 2021	0.96 (0.65 – 1.42)	0.83	0
Nasasra et al. 2020	1.15 (0.81 – 1.61)	0.43	0
Yu et al. 2019	1.08 (0.76 – 1.54)	0.67	0
**Major bleeding events in the follow up time**			
CruzGonzález et al. 2020	1.68 (1.17 – 2.42)	0.005	0
Dai et al. 2021	1.61 (1.14 – 2.25)	0.006	0
Freixa et al. 2016	1.65 (1.15 – 2.36)	0.006	0
Freixa et al. 2021	1.72 (1.17 – 2.51)	0.005	0
Mohrez et al. 2021	1.69 (1.12 – 2.54)	0.01	0

Nasasra et al. 2020	1.60 (1.13 – 2.27)	0.008	0
Yu et al. 2019	1.55 (1.08 – 2.23)	0.02	0
